# Epigenetic Mechanisms of Senescence in Plants

**DOI:** 10.3390/cells11020251

**Published:** 2022-01-12

**Authors:** Matin Miryeganeh

**Affiliations:** Plant Epigenetics Unit, Okinawa Institute of Science and Technology Graduate University, 1919-1 Tancha, Onna-son, Okinawa 904-0412, Japan; matin.miryeganeh@oist.jp

**Keywords:** plant senescence, aging, epigenetic, DNA methylation, histone modification, chromatin remodeling, miRNA

## Abstract

Senescence is a major developmental transition in plants that requires a massive reprogramming of gene expression and includes various layers of regulations. Senescence is either an age-dependent or a stress-induced process, and is under the control of complex regulatory networks that interact with each other. It has been shown that besides genetic reprogramming, which is an important aspect of plant senescence, transcription factors and higher-level mechanisms, such as epigenetic and small RNA-mediated regulators, are also key factors of senescence-related genes. Epigenetic mechanisms are an important layer of this multilevel regulatory system that change the activity of transcription factors (TFs) and play an important role in modulating the expression of senescence-related gene. They include chromatin remodeling, DNA methylation, histone modification, and the RNA-mediated control of transcription factors and genes. This review provides an overview of the known epigenetic regulation of plant senescence, which has mostly been studied in the form of leaf senescence, and it also covers what has been reported about whole-plant senescence.

## 1. Overview

Senescence (from the Latin word “senescere”, meaning to grow old) is the final, main developmental phase transition in plants. It occurs at different levels, including at the level of cells, tissues, organs, and the whole plant [1]. Senescence plays an important role in photosynthesis, nutrient remobilization, the completion of the plant life cycle, successful reproduction, stress responses, adaptability, and fitness [2]. Thus, as a major developmental stage, the initiation, progress, and termination of senescence are regulated by complex regulatory pathways and multiple levels of machinery that are influenced by both internal and external factors [2,3]. Functional genetic and transcriptomic studies have reported that, upon senescence, genetic reprogramming changes the expression of many senescence-related genes [4]. For instance, time series analyses on aging *Arabidopsis* leaves have shown changes in the expression of up to 16% of genes during senescence [4,5]. Furthermore, the expression of the genes encoding for those transcription factors also changes during senescence, which makes the mechanisms behind senescence even more complex [3]. Genes that are upregulated during senescence (e.g., genes for the degradation and recycling of nutrients) are called senescence-associated genes (SAGs), and those downregulated (e.g., genes for photosynthesis and chloroplast development) are named senescence downregulated genes (SDGs). Transcription factors (TFs), such as NAC (NAM, ATAF and CUC) and the WRKY family, are known as key TFs that act upstream of the senescence regulatory pathways to activate the senescence genes [1,4,6].

In addition, epigenetic mechanisms that are known to play an important role in regulating the transition of plants to the next developmental stages also have a key impact on senescence [7]. They dynamically alter the chromatin structure at certain loci, which makes the DNA accessible (euchromatin) or inaccessible (heterochromatin) for TFs and thereby activate or inactivate the senescence-related genes, respectively [2]. Studying the expression of epigenetic genes during senescence could help to understand if senescence is associated with changes in the epigenetic pattern (e.g., DNA methylation) of those genes. For instance, *RD20* (responsive to dehydration-20) and *RAP2.4* (related to *AP2.4*) are *Arabidopsis* drought stress-responsive genes which are also upregulated during leaf senescence, and their expression is controlled via differential methylation patterns [4]. Using published gene expression data [4], the expression pattern of genes that are known (or suggested) to be involved in DNA methylation [8] during senescence was studied [9], and up to 23% of the examined genes showed differential expression at different stages of the aging of *Arabidopsis* leaves.

**Figure 1:**
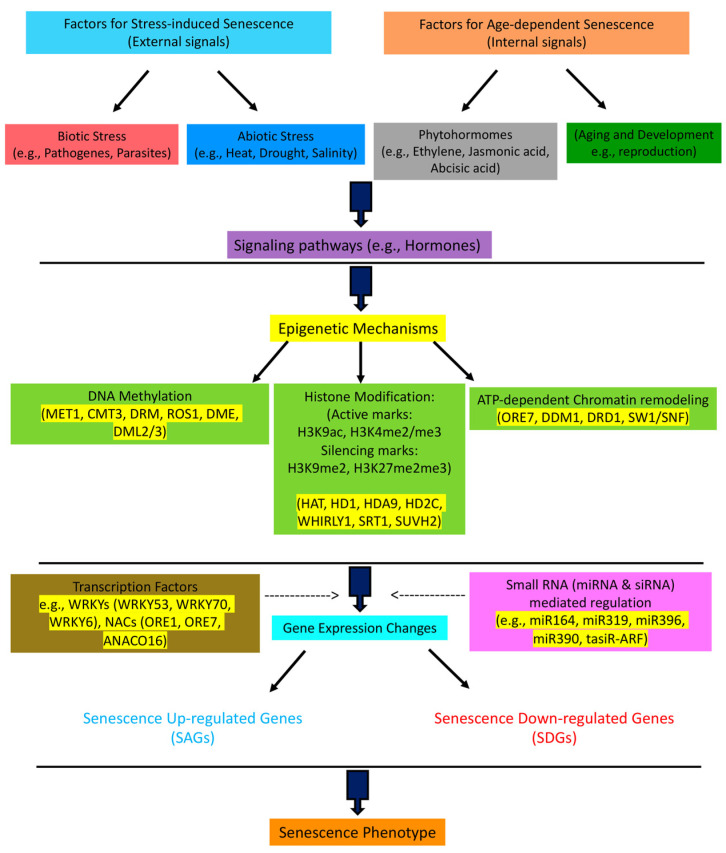
Multilayer regulatory network of plant senescence. The senescence program is influenced by external (stress-induced) and internal (developmental) signals. It is known that downstream of signaling pathways, such as in phytohormone activity, and upstream of transcription factors, such as WRKYs and NACs that regulate the expression of senescence genes, there are higher-level epigenetic mechanisms that control the process of senescence. The epigenetic mechanisms regulate the expression of transcription factors and other senescence-related genes via DNA methylation, histone methylation/acetylation, chromatin remodeling, and the interactions of small RNAs, such as miRNA and siRNA, with senescence-related genes. The name for the main factors in every step is given.

It should be noted that most senescence studies of plants have focused on *Arabidopsis* leaves, which have a very short lifetime, and their senescence may not necessarily—or at least entirely—be linked to the whole-plant senescence. Whole-plant senescence leads to death, and it depends on both chronological age and environmental cues, and identifying the changes in gene expression and epigenetics during leaf senescence may or may not be directly relevant to what happens at the time of whole-plant senescence [10]. This review aims to summarize the reported epigenetic mechanisms that regulate plant senescence either at the level of leaf senescence or whole-plant senescence when available (Figure 1). However, future studies focusing on whole-plant senescence are needed to clarify the molecular mechanism (genetic or epigenetic) behind the final senescence of a fully developed, aged plant.

## 2. DNA Methylation and Plant Senescence

DNA methylation in plant genomes is more extensive than in animals [11], and it might be either symmetric, as with CG, which is mainly modulated by DNA- METHYLTRANSFERASE-1 (*MET1*), or CHG (H is A, T or C), which is mainly modulated by CHROMOMETHYLASE 3 (*CMT3*), or asymmetric, as with CHH DNA-methylation, which is usually controlled by DOMAINS-REARRANGED METHYLTRANSFERASEs (*DRMs*) [11]. DNA methyltransferases methylate the cytosine bases that make 5-methylcytosine. The methyl groups in DNA can be removed by demethylating enzymes, such as REPRESSOR OF SILENCING 1 (*ROS1*), DEMETER (*DME*), and DEMETER-LIKE proteins (*DML2/3*) that contain DNA glycosylase domains [12,13]. DNA methylation can regulate gene expression by changing the methylation status of promoters or coding regions of the genes, which changes their binding ability in relation to transcriptional factors [14]. In addition, DNA methylation may occur at repeats, such as transposable elements (TEs), to stabilize the heterochromatic structure through silencing those repeats [15].

Global DNA methylation changes that occur across developmental life events are reported in plants, such as the giant redwood tree [16]. The effect of DNA methylation on the process of senescence is also reported in various aspects, including genes encoding methylase and demethylase enzymes, such as *MET1, CMT3, ROS1, DME* and *DML2/3*, but the specific mechanisms are not clear yet [17]. In a study on maize, it was suggested that DNA methylation may play a role in whole-plant senescence, as DNA methylation changes were observed at life event transitions that silenced the MuDR (Mutator-–Don Robertson) transposable elements [18]. Studies on *MET1* transgenic *Arabidopsis* plants have reported that when *MET1* activity is reduced, for example in *met1* mutants or with the constant expression of the *MET1* antisense gene, hypomethylation in genomic DNA and developmental abnormalities happen. When studying transgenic plants with a *MET1* antisense gene fused to the *DEMETER (DME)* promoter (*DME:MET1 a/s*), Kim et al. [19] showed that when *MET1* expression is suppressed, major delays in senescence and other developmental deficiencies occur [19].

In addition, the involvement of DNA methylation in maintaining genome integrity by silencing TEs and repetitive sequences during senescence has been reported before [20,21]. Studies on *Arabidopsis* and barley have shown the release of TEs during leaf senescence [22,23]. He et al. [24] detected a retrotransposon, called “NMR19” (naturally occurring DNA methylation variation region 19), and its location in the genome, and found that the status of its methylation varies among different *Arabidopsis thaliana* ecotypes. A class that they named NMR19-4 appeared to be a naturally occurring epiallele that controlled leaf senescence. They found that the DNA methylation of NMR19-4 negatively regulates the transcription of the gene “pheophytin pheophorbide hydrolase (PPH)”, which encodes an enzyme involved in chlorophyll breakdown during leaf senescence. The levels of DNA methylation and TEs were also shown to be related [24]. Changes in the level of DNA methylation have been shown during aging in angiosperms as well [25]. In another study, Trejo-Arellano et al. [26] investigated global methylation levels in *Arabidopsis*’ dark-induced senescent leaves and reported that, upon senescence, chromatin-silencing genes were downregulated, which led to the interruption of TE silencing and the reactivation of young TEs. They also found that although heterochromatin at chromocenters was decondensed, the global DNA methylation pattern was maintained, with only localized changes in CHH methylation. They concluded that senescence is associated with global chromatin reorganization but is only limited to changes in localized DNA methylation. Vatov et al. [27] observed faster senescence progression in two methylation mutants (*ros1* and the triple *dmr1/2 cmt3* knockout). They showed that as senescence progressed, wide-type plants showed a moderate decrease in DNA methylation, mostly in a CG context, and the most senescent leaf was mainly associated with CHH de novo methylation.

In some plants, aging has shown to cause the loss of morphogenic ability to the point that mature plants do not have the ability to propagate vegetatively [28]. It has been reported that this loss of morphogenic ability during plant senescence is related to gene expression changes modulated by DNA methylation [29]. Fraga et al. [30] investigated the differences in the extent of DNA methylation among the developmental stages of the *Pinus radiata* trees and found significant DNA methylation differences between the meristematic tissues of juvenile and mature trees, while they found little differences in DNA methylation between their differentiated tissues. They reported a gradual reduction in the extent of genomic DNA methylation in meristematic areas as the level of reinvigoration increased and, therefore, they suggested that the level of DNA methylation can serve as a marker of aging and reinvigoration.

The de-methylation of DNA has also been reported to be accompanied by aging. Studying *A. thaliana* plants as they aged, Ogneva et al. [31] reported the reduced expression of methyltransferase genes, *CMT3* and *METI*, and consequently decreased the cytosine methylation of DNA regions with aging, while the transcription of demethylase genes, *ROS1*, *DME*, *DML2* and *DML3* increased. They concluded that plants experience the demethylation of DNA during aging through a reduction of DNA methyltransferase and an increase in levels of demethylase enzymes [31]. Yuan et al. [32] also showed that DEMETER-like DNA demethylase gene *DML3* controls leaf senescence as the *dml3* knockout mutants showed enriched DNA methylation in the promoters of many *SAG*s that suppressed their expression, resulting in delayed leaf senescence. They suggested that *DML3*-mediated DNA demethylation may control leaf senescence by regulating the expression of some *SAG*s [32].

## 3. Histone Modifications and Plant Senescence

Studies have shown that besides DNA methylation, various types of histone modifications also change the dynamic expression of genes when plants move through their developmental phases, such as senescence, or as they respond to the environmental signals. The DNA of eukaryote organisms is wrapped around eight histone molecules (a histone octamer), forming the nucleosome, which is the element of the chromatin structure [33]. Nucleosomes can be disassembled/reassembled at specific genome locations in response to environmental and/or developmental cues. The amino acids at the histone N-tails protruding from the histone octamer can be modified by different post-translational modifications, such as methylation, acetylation, H2B monoubiquitination, and phosphorylation, which change the structure of chromatin and, therefore, the expression of the genes, as the interaction of DNA–histone and the accessibility of transcription factors change [34]. Among the different histone modifications, the acetylation of lysine 9 at histone H3, (H3K9ac) and the tri-methylation of lysine 4 at histone H2 and H3 (H3K4me2/me3) are associated with inducing the transcription of the genes, while the di-methylation of lysine 9 at histone H9 (H3K9me2) and the tri-methylation of lysine 27 at histone H2 and H3 (H3K27me2/me3) marks are involved in repressing the transcription of the genes [35,36]. The main histone modifiers are histone acetyltransferases (HATs), histone deacetylases (HDAs or HDs), histone methyltransferases, and histone demethylases [7,37]. Chromatin remodeling via histone modification has been reported to be a key regulatory mechanism of plant senescence [38,39]. However, the exact mechanism of histone modification and chromatin-remodeling enzymes regulating senescence is not clear yet. The relationship between histone modification and the expression of senescence-related genes has been reported mostly for H3 histone in the forms of active marks (e.g., H3K4me2/me3 and H3K9ac) and silencing marks (e.g., H3K27me2/me3) [17]. The active histone H3K4me3 is more common than H3K9ac, and the expression of more senescence-related genes is associated with H3K4me3 levels [40].

One of the first studies that showed a direct connection between histone modification and leaf senescence regulation was when Ay et al. [39] reported the involvement of the SUPPRESSION(VAR)3-9 homolog2 (*SUVH2*) histone methyltransferase in H3 lysine methylation and its role in the delay of leaf senescence. They showed that in plants with overexpressing *SUVH2* histone methyltransferase, which is involved in RNA-directed DNA methylation and transcriptional gene silencing by keeping the chromatin structure compact, leaf senescence is delayed. The delay in senescence was because of the repression of key senescence regulators, such as *SIRK* (senescence-induced receptor-like serine/threonine-protein kinase) or *SAG101* (senescence-associated carboxylesterase 101) and was connected to the inhibition of *WRKY53*, a main transcription factor that promotes leaf senescence. The levels of H3K27me2 and H3K27me3 at the 5′-end region of *WRKY53* was elevated and therefore caused the suppressed transcription of WRKY53 along with some SAGs [39,41]. Jing et al. [42] also reported that the expression of SUVH2 causes the repression of *WRKY53* and some of the SAGs through H3K27me2/3 modifications. The overexpression of SUVH2 histone methyltransferase represses almost half of the senescence-related regulatory factors (SRRFs). It was reported that in plants with overexpressing SUVH2, leaf senescence is delayed by about two weeks and, therefore, SAGs are either not expressed or repressed [9]. Epigenetic indexing at the *WRKY53* locus showed that complex epigenetic processes are involved in senescence-related gene expression reprogramming. The induced expression of *WRKY53* during senescence was associated with an increasing number of active histone marks (H3K4me2 and H3K4me3) at the 5′ end and coding regions of WRKY53 [39], similar to many SAGs, that their upregulation during senescence is associated with increased H3K4me3 levels as well [43]. The level of H3K4me3 was increased during either dark-induced or developmental (age-dependent) *Arabidopsis* leaf senescence, mostly within *WRKY53*, regulating the expression of many SAGs [9].

Brusslan et al. [43] studied genome-wide changes in an active (H3K4me3) and silencing (H3K27me3) histone marks using mature and senescing *Arabidopsis* leaves, and found SAGs with higher H3K4me3 signals in the older leaves, while for genes that downregulate during senescence (SDGs), H3K4me3 was higher in the younger leaves. Likewise, the silencing histone mark, H3K27me3, was lost at some SAGs in the older leaves, and established at some SDGs. This again indicated the role of epigenetic regulation in the expression of senescence-related genes during leaf senescence [43]. Next, in a genome-wide distribution study of H3K4me3 marks at different time points during natural developmental senescence, the researchers found upregulated genes with higher H3K4me3 in older leaves, confirming the important role of H3K4me3 in senescence [40]. They again reported that in senescing leaves, the H3K4me3 mark increased in senescence upregulated genes and decreased in senescence downregulated genes. In addition, genes that upregulate at senescence time showed a loss of the H3K27me3 mark in older tissue, while only a few of the senescence downregulated genes gained the H3K27me3 mark [40].

A continuous lack of light causes changes in the expression of many genes, which leads to various developmental disruptions, including early leaf senescence [44]. To investigate the epigenetic mechanism behind this, Yan et al. [45] studied the global epigenomic profiles of H3K4me3 under dark stress in *Arabidopsis*. They found an increase in the number of H3K4me3 marks after three days of darkness, and the genes with dark-increased H3K4me3 were mainly involved in senescence. The upregulated genes after dark treatment were highly expressed in senescent leaves as opposed to younger leaves and, likewise, the downregulated genes after dark treatment showed lower expression in senescing leaves than in younger leaves [45]. They also compared the changes in H3K4me3 in dark-induced and age-associated leaf senescence, where they found that H3K4me3 changes were correlated with gene expression during age-related leaf senescence, as it had also been reported by Brusslan et al. [40]. GO enrichment analysis showed that some of the upregulated genes with increased H3K4me3 signals were genes, such as *WRKY6*, *SAG113*, and *SAG101*, that are known to promote senescence, and some were involved in dark-induced leaf senescence (e.g., Abscisic acid (ABA) Insensitive 5 (*ABI5*), ETHYLENE INSENSITIVE 3 (*EIN3*), and ORESARA 1 (*ORE1*)) [46]. Therefore, they concluded that H3K4me3 has an important impact on the regulation of SAGs and there are overlapping genes with changed H3K4me3 signals during dark-induced and natural leaf senescence. *JMJ16* is an *Arabidopsis* JmjC-domain-containing protein and acts as an H3K4 demethylase. A decrease in *JMJ16* is reported to be associated with the increase in the amount of H3K4me3 during senescence [47]. Liu et al. [47] reported that *JMJ16* negatively affects age-dependent leaf senescence by repressing *WRKY53* and *SAG201* via its demethylase activity and reducing their level of H3K4me3. They found the overexpression of various SAGs associated with the hypermethylation of H3K4me3 in loss-of-function *jmj16* mutants [47].

An important role of leaf senescence is known to be facilitating nutrient remobilization to younger leaves and reproductive organs to maximize fitness, and many SAGs are upregulated during the process. It is interesting to know how SAGs remain transcriptionally inactive before the onset of leaf senescence to ensure photosynthesis. Wang et al. (Wang, Gao et al. 2019), identified an epigenetic mechanism that prevents the premature expression of these genes. They reported that RELATIVE OF EARLY FLOWERING 6 (REF6) promotes H3K27me3 demethylation at the promoter and coding regions of ten target senescence genes to activate them. The number of H3K27me3 marks decreases during senescence, as they repress the expression of senescence genes. This is caused by genes, such as REF6 [48]. REF6 directly activates senescence regulators, such as ETHYLENE INSENSITIVE 2 (EIN2), ORE1, and NAP (NAC-like, activated by AP3/P1), and acts as a binding protein for the promoter of NYE1 (NONYELLOWING1) gene and promotes chloroplast degradation during leaf senescence by upregulating this gene [49].

Another histone mark that is reported to have a role in regulating leaf senescence is histone acetylation (H3K9ac). The coordinated activities of histone acetylation and deacetylation play important roles in gene expression and are organized by HATs and HDAs enzymes. While histone acetylation correlates with active transcription, histone deacetylation is often associated with the repressing and silencing of the gene by removing acetylation and inducing chromatin compaction [50,51]. The direct effect of histone acetylation on the expression of the genes that may promote senescence was first reported in a study on an acetyltransferase Elongator. Zhu et al. [52] studied the function of an Elongator complex protein 2-like gene in tomatoes via RNAi-mediated gene silencing and found that the silencing of this acetyltransferase Elongator gene accelerated leaf senescence and sepal senescence. Brusslan et al. [40] analyzed the abundance of both H3K4me3 and H3K9ac in *Arabidopsis* leaves at different time points during developmental senescence and reported that many upregulated senescence-related genes were also marked with H3K9ac, which activates TFs, such as *WRKY53*. They found that the H3K9ac levels around SAGs were high at the early stages of senescence and decreased gradually as senescence progressed. This was the opposite of what happens in the case of the H3K4me3 level, which it is usually low at the early stage of senescence and increases drastically towards the end of the senescence stage. The histone marks were highly convergent throughout aging; however, the average number of H3K4me3 marks around the SAGs covered the gene area almost two times more than what was covered by H3K9ac marks.

Histone deacetylation has been reported as a leaf senescence regulator in *Arabidopsis* as well. Huang et al. [53] found that *HDA15* interacts with the single-stranded DNA-binding protein *WHIRLY1* and increases H3K9ac in the promoter of *WRKY53* to suppress its expression and therefore delay leaf senescence. Furthermore, histone acetyltransferase *HAC1* is known to positively regulate leaf senescence [54], whereas various HDACs, including *HDA9*, *HDA15*, *HD2C*, and *AtSRT1* (from SILENT INFORMATION REGULATOR2 (*SIR2*) family proteins), have been shown to have a negative effect on stress-triggered senescence in *Arabidopsis* [55,56]. Analyzing the functions of histone deacetylases (HDAs), Tian and Chen [57] studied transgenic plants that overexpress antisense *HDA19*, a histone deacetylases also known as *AtHD1* that belongs to the RPD3 (reduced potassium dependency 3) class of histone deacetylases [37], and reported the global changes in the histone deacetylation profile that affected the regulation of senescence. Wu et al. [58] showed that the *loss*-*of*-*function* mutants of *HDA6* show increases in the acetylation of histone H3 and the downregulation of *SAG12* and *SEN4*, which delayed the senescence. However, the expression of *RPS17* (ribosomal protein S17), which is normally downregulated at the time of senescence, was maintained at high level in the mutants. *HDA9* is known to promote leaf senescence and regulate the genes involved in the onset of both developmental and dark-induced senescence. In *HDA9* mutants, leaf senescence is reported to be delayed [51,59]. Chen et al. [51] reported the mechanism by which the HDA9-PWR-WRKY53 complex coordinates several signaling pathways to regulate the global transcription of genes during leaf senescence. They showed that *HDA9* makes a complex with a SANT domain-containing protein POWERDRESS (PWR) and the transcription factor *WRKY53*. The WRKY53 then directs POWERDRESS and HDA9 to the promoters of negative senescence regulators, such as *AUTOPHAGY 9* (*ATG9*), *NUCLEAR PROTEIN X 1* (*NPX1*), and *WRKY57* [38]. As mentioned before, most of these senescence studies have focused on *Arabidopsis* leaf senescence, which has a short life and can be easily experimentally manipulated. Future studies focusing on whole-plant senescence are necessary to obtain a better understanding of the role of histone modifications in plant senescence. One study has focused on the epigenetic and transcriptional mechanisms of fruit senescence in longan trees, which showed that histone deacetylase HD2 interacts with ethylene response factors (*ERFs*) and is involved in longan plant fruit senescence [60]. They analyzed one histone deacetylase 2-like gene, *DlHD2* and two ethylene-responsive factor-like genes, *DlERF1* and *DlERF2*, during fruit senescence, and showed that *DlHD2* might cooperate with *DlERF1* to regulate the transcription of fruit senescence genes [60].

## 4. ATP-Dependent Chromatin Remodeling and Senescence

In addition to histone modifications and DNA methylation, ATP-dependent chromatin reorganization modulates the DNA accessibility for transcription factors and RNA polymerase II, and therefore regulates the transcription of the genes within the chromatin structure [61,62,63]. This dynamic change of DNA accessibility influences many processes in plants, including developmental events, such as flowering time and senescence, as well as the response to stresses and environmental changes [64,65]. Studies have shown that changes in the chromatin structure during senescence by chromatin-remodeler enzymes play an important role in controlling leaf senescence [17]. During leaf senescence, photosynthesis genes are downregulated while SAGs are sequentially upregulated. The process recruits dynamic epigenetic mechanisms with the regulation of chromatin reorganization, where modified histone marks participate in ATP-dependent chromatin remodeling [66].

Heterochromatic and euchromatic areas bear different histone marks. The heterochromatin region at the pericentric area is characterized by typical heterochromatic histone marks, such as H3K9me2, and the euchromatic regions bear marks, such as H3K4me2 and H3K4me3. Therefore, it is feasible to study the changes in distribution of eu- and heterochromatic marks in the nuclei during developmental transitions using electron microscopy. For example, Ay et al. [39] showed the re-organization of hetero- and euchromatic regions of nucleus in senescing cells and their association with the dense chromocenters [39]. The heterochromatic regions that are primarily transcriptionally inactive were distinguished during leaf senescence from the surrounding euchromatic areas, which are less transcriptionally active. However, further studies are needed to understand whether the de-condensation of chromatin at centromeric and pericentric regions at the onset of leaf senescence is an indirect event caused by nuclei disintegration, or if it is a planned process for plants to enter the senescence phase [9].

The overexpression of a chromatin remodeler AT-hook protein ORESARA 7 (*ORE7*) has been reported to cause a strong delay in leaf senescence [67]. The delayed senescence was accompanied with the late expression of SAGs, suggesting that senescence is affected by the AT-hook protein through the reorganization of the chromatin structure. ORE7 inhibits chromatin de-condensation and, therefore, prevents the access of senescence transcription factors. Other chromatin-modifying proteins that are reported to be involved in the expression of senescence-specific genes are *SNF2* (SUCROSE NONFERMENTING) family genes, such as *CHR10* (*ALTERED SEED GERMINATION 3* (*ASG3*)) and *CHR19* (*ETL1*), which are shown to be upregulated during senescence [4]. Members of the SWI/SNF family chromatin remodelers are another group of proteins reported to upregulate SAGs, but the exact mechanism of this is not known. *REF6* facilitates the recruitment of an SWI/SNF chromatin remodeling ATPase *BRAHMA* (*BRM*), which directly targets many senescence-related genes [17,68]. The *BRM* can also colocalize senescence genes with *REF6*, meaning *BRM*-containing SWI/SNF complexes might act along with *REF6* to epigenetically control the leaf senescence by controlling the chromatin structure of senescence genes [17,68], and the dysfunctional mutants of BRM have been shown to accelerate leaf senescence [69,70]. Another gene silencer from the SWI2/SNF2 subfamily is chromatin remodeling protein 1 (*DRD1*: *DEFECTIVE IN RNA-DIRECTED DNA METHYLATION 1*) that is reported to be involved in the regulation of leaf senescence genes [71]. Studying the mutants of chromatin-remodeling genes, *DRD1* and *DDM1*: *DECREASED DNA METHYLATION 1* (ATP-dependent DNA helicase *DDM1* from the same group of proteins as *DRD1*), Cho et al. [71] found that mutants of these genes cause the repression of SAGs and delayed leaf senescence. They reported that after dark treatment, photosynthetic parameters, including chlorophyll content, were lower in control plants than *drd1-6* mutants, and the expression of SAGs was significantly inhibited in the *drd1-6* mutant even after 5 days of dark-induced senescence [71]. To compare epigenetic changes after dark-induced senescence, they analyzed the expression levels of centromeric (*CEN*) and transcriptionally silent information (*TSI*) repeats in both wild type (WT) and the *drd1-6* mutant, which showed a much lower increase in the number of mutants. They concluded that SWI2/SNF2 chromatin-remodeling proteins play a key role in the regulation of leaf senescence through epigenetic regulation. The histone deacetylase and *ore7* mutants have also been reported to delay flowering, which might suggest that they cause delays in the entire developmental program [7]. Future analyses are needed to investigate if and how the encoded proteins are involved in chromatin remodeling during senescence.

## 5. Stress-Induced vs. Age-Dependent Senescence

Many of senescence-associated genes also play a role in other biological processes. This suggests that genes that have evolved for senescence may have been recruited in other biological networks as well [72]. For example, senescence regulatory pathways have shown similarity to the regulatory pathways of plant defense and stress responses. In addition, the regulatory pathways of age-dependent (developmental) senescence are reported to interact with the senescence regulatory pathways that are triggered by environmental stresses [1]. There have been reports of overlap between the genetic programs that underlie these two senescence paths and cross-talk between them optimizes the proper senescence timing for plants [73]. For example, ORESARA1 (*ORE1*), a key NAC TF that promotes developmental leaf senescence, is also implicated in salt-triggered senescence [74,75]. Dark treatment and biotic stresses also have shown to change the regulation of senescence genes and induce senescence [75,76]. However, the overlap between the signaling pathways of programmed and induced senescence is not well understood. Guo and Gan [22] studied the effect of 27 senescence-promoting stresses on the transcription of senescence genes and found that after long-term stress treatment, the signaling pathway during senescence progression overlaps between induced and programmed senescence; however, only small similarity was found at the beginning of stress treatment, suggesting that the initial stage of senescence is probably very specific.

Heterochromatic patterns are shown to change during senescence as they do in response to stress, too [77]. Scientists have discussed that both plant stress response and leaf senescence are regulated by chromatin changes and are under the control of similar epigenetic mechanisms [7,9], which implies that the epigenetic regulation of the main factors involved in these two processes could function as a fine-tuning of the different gene responses. For instance, the H3K4me3 patterns and the expression of *WRKY70*, which encodes a TF involved in the response to salicylic acid and jasmonic acid, and also in leaf senescence [78], is shown to be controlled by HMT ARABIDOPSIS HOMOLOG OF TRITHORAX1 (*ATX1* histone methylase) [79]. The transcription factors *WRKY6* and *WRKY53* also are known to be involved in the regulation of both senescence and responses to pathogens [80,81]. Studies have shown that besides the regulation of senescence-related genes, H3 and H4 histone modifications associate with the transcription of stress response genes in plants as well [39,82,83,84,85]. Furthermore, in the *hda19* mutants, genes that are in charge of both plant developmental processes, including flowering and senescence, and stress responses are differentially expressed. This shows that the regulatory pathways of the main developmental phases, such as flowering and senescence, and of biotic and abiotic stress responses, are connected, and together orchestrate a network controlling environment-sensitive plant development, in which epigenetic mechanisms seem to be involved in forming a higher-order control level [9]. In addition, it has been shown that biotic and abiotic stresses, such as pathogen attack and high salinity, could be linked to the activation of TEs, and also changes in the pattern of DNA methylation. Therefore, it is possible that stress-induced senescence may be connected to changes in DNA methylation, and TE activities which could then affect the expression of neighboring genes [9].

*WHIRLY1* is a chloroplast–nucleus protein that has a role in regulating leaf senescence through binding to the promoters of SAGs, such as *HvS40*. Studying the expression of *WHIRLY1* in premature senescence triggered by drought stress, Janack et al. [84] reported delayed senescence in transgenic barley plants with an RNAi-mediated silenced HvWHIRLY1 gene, which confirmed the role of this gene in response to stress-induced senescence. Although drought treatment resulted in rapid leaf senescence in wide-type plants, WHIRLY 1 knockdown lines (RNAi-W1) remained green, and the expressions of both senescence-associated and drought stress-responsive genes were delayed. In addition, even though wild-type plants showed increased levels of H3K9ac, of the *HvS40* gene, under drought stress, indicating the role of epigenetic mechanisms in response to drought stress, no significant increase in H3K9ac was seen in RNAi-W1 transgenic plants (which are impaired in their accumulation of WHIRLY1). They suggested that *WHIRLY1* knockdown results in delayed senescence that involves changes in gene expression, by means such as altering the chromatin structure via histone modification [84].

Abscisic acid (ABA) is a stress- and senescence-associated phytohormone which is reported to trigger the signaling involved in changes in histone acetylation levels [86,87]. On the other hand, studying the effect of dark stress on barley leaves, Becker and Apel [88] showed increasing levels of three mRNAs, from which two were also induced by other stresses, such as wounding, drought stress, jasmonate, and abscisic acid (ABA), but not in naturally senescing leaves, suggesting that they may be specifically induced by stress, rather than senescence. Therefore, it is not easy to distinguish natural senescence regulatory pathways from stress responses, as the molecular mechanisms behind senescence overlaps with those of plant defense. Further studies using epigenetic marks might help to separate these two processes more clearly [17].

## 6. DNA Damage, Aging, and Epigenetics

DNA damage, either caused internally, for example from replication errors, or externally from genotoxic stresses [89], abiotic stresses, and metabolic by-products (such as reactive oxygen species (ROS)) [90], is unavoidable for plants during their lifetime. DNA double-strand breaks (DSBs) may result in deleterious mutations and premature aging and senescence [91]. Thus, plants need to locate and repair the damaged DNA in order to maintain the genome stability and integrity that is vital for their survival [92], and that for which they have evolved a network of DNA repair pathways [93]. Plants not only have orthologues of most DNA damage repair (DDR) components found in animals, including *ATM* (ATAXIA TELANGIECTASIA MUTATED) and *ATR* (ATM AND RAD3-RELATED), two main protein kinase activated in response of DSBs and SSBs (single-strand breaks), respectively [94], but they also own various plant-specific DDR components, such as SUPPRESSOR OF GAMMA RESPONSE 1 (SOG1) [95].

Chromatin remodeling, such as histone modification, is reported to be involved in the repair of DSBs in animals [96]. In *Arabidopsis*, the abundance of acetylation and methylation in histone H3 is shown to respond to X-ray radiation [97]. Therefore, histone modifications seem to be a common epigenetic response to DNA damage in both animals and plants. However, whether double-strand DNA breaks may induce premature aging in plant and affect their lifespan, as it does in animals [91], is not well understood. In their study, Li et al. [98] provided evidence that the balance between the DSB and double-strand repair (DSR) may define the rate of aging in plants. They reported the increased accumulation of DSBs, likely caused by the decreased efficiency of DNA repair with age, as one of the main identifiers of aging in plant leaves, which is consistent with the DNA damage theory of aging [99]. In addition, they showed an important role for histone methylation in regulating bleomycin (BLM)-induced leaf senescence. They monitored the senescence phenotypes in *loss-of-function Arabidopsis* mutants for key members of the DNA repair pathway and reported that the deficiency in *ATM*, which is the main transducer of the DSB signal, results in the age-dependent accumulation of DSBs and leads to premature senescence. This is epigenetically regulated through the inhibiting of the transcription of senescence-associated TFs, such as *ANAC016* (NAC domain-containing protein 16), *WRKY6*, *WRKY53*, and WRKY75, by *ATM*, which then manifests as leaf senescence when SAGs are upregulated by the modulation of H3K4me3/H3K27me3 marks [98]. They also reported that SUVH2 functions downstream of ATM in DSB-induced leaf senescence of *Arabidopsis* [98]. They suggested that DSBs are an evolutionarily conserved promoter of the aging processes in plants. Further studies are needed to investigate the molecular mechanisms behind the involvement of ATM and SUVH2 in regulating the histone methylation of *SAG*s during DSB-induced leaf senescence.

## 7. Small RNAs and Senescence

Small RNAs (sRNAs) consist of about 20–30 nucleotides and have been reported to regulate the expression of the genes involved in plant development and stress responses [100,101]. They include microRNAs (miRNA) and small interfering RNAs (siRNAs) that bind to target mRNAs and inhibit their translation. In addition to the 21 nucleotide miRNAs, plants generate 24 nucleotide miRNAs that bind to mRNAs and call the de novo DNA methylation machinery for the methylation of adjacent DNA sequences [102]. They may also interact with DNA methylation for RNA-directed DNA methylation (RdDM), which prevents transposon activity [103].

Some miRNAs and siRNAs are reported to regulate the progress of plant senescence by controlling the expression of TFs or phytohormone response factors [3,7]. For example, the complex network comprising of *ORE1*, *miR164*, and *EIN2* seems to robustly regulate age-induced cell death in *Arabidopsis* leaves. It has been reported that miR164 in *Arabidopsis* negatively regulates the expression of *ORE1*, which is a positive regulator of leaf senescence [104]. Kim et al. [104] showed that as the expression of miR164 decreases towards senescence via the negative regulation of *EIN2*, it leads to the increased expression of *ORE1*, which promotes age-induced cell death in *Arabidopsis* leaves. Therefore, in the *miR164* mutant, early senescence occurs. *miR319* and *miR396* were also identified as positive regulators of senescence because they repress members of signaling pathways (*miR319*—DNA-binding transcription factor) and induce a local limitation of regulator activity (miR396—growth-regulating factor, GRF) [104,105,106].

It has been shown that in *Arabidopsis arf2* (Auxin response factor 2) mutants, leaf senescence is delayed [107,108]. *ARF2* is involved in auxin-dependent senescence, and it can be knocked out by the *miR390*-mediated production of a transcriptional silencing complex protein (TAS3). Phased, secondary, small interfering RNA (phasiRNA) is a class of plant-specific non-coding siRNA with a phase alignment structure, mediated by miRNAs at protein coding sites and non-coding sites (collectively called phasiRNA-producing loci, *PHAS* loci) [109]. A group of these siRNAs are the *trans*-acting siRNAs (tasiRNAs). TAS3 (*TRANS-ACTING SIRNA LOCUS 3*) encodes trans-acting siRNAs and suppresses the transcription of the auxin response genes, such as *ARF2* [110], which is a repressor of auxin responses that are involved in the timing of senescence. Therefore, TAS3 may negatively control plant senescence via *ARF2* repression.

In another study, Schommer et al. [105] reported that the regulation of senescence by *miR319* targets where the overexpression of *miR319* led to the generation of a stay-green phenotype [105]. They identified a process controlled by the *miR319*-regulated clade of *TCP* (*TEOSINTE BRANCHED/CYCLOIDEA/PCF*) TF genes by studying the downstream events regulated by five TCP-TFs that are controlled by the *miR319* in *A. thaliana*. They analyzed leaf extracts from plants with increased activity of miR319 and found that the expression changes of the biosynthetic genes result in changed jasmonic acid levels and that changes in senescence behavior return to normal after treating with jasmonate. They concluded that *miR319*-controlled TCP transcription factors may negatively regulate leaf growth and positively regulate leaf senescence. Luan et al. [111] reported that ABA in tomatoes (*Solanum lycopersicum*) can improve the plant’s tolerance to various stresses by changing the expression and interaction of some miRNAs, and phasiRNAs and their target genes. As ABA is known to accelerate senescence as well, future studies investigating the potential role of small RNAs in this process could expand our understanding of the importance of small RNAs in senescence.

## 8. Conclusions

Senescence is a very important developmental phase in plants which has to be in coordination with other developmental phases, and it also needs to synchronize with environmental signals [112]. In plants, the switch from one stage to the next (e.g., vegetative to reproductive) is often connected to the time of whole-plant senescence and vice versa [9]. This orchestrated interplay is necessary for maximizing plants’ fitness and is controlled by multi-level regulatory mechanisms [10]. Besides *cis*- and *trans*-acting factors that activate the transcription of SAGs, it has been reported that epigenetic mechanisms can regulate plant senescence via DNA methylation, histone modification, ATP-dependent chromatin remodeling, and non-coding RNAs [9], and different epigenetic regulation mechanisms are known to interact with each other as a complex network [113,114,115]. Senescence might occur either in the age-dependent or stress-induced manner, and most plant senescence studies have focused on leaf senescence. Dark treatment has commonly been used to manually trigger senescence. Other environmental stresses, such as extreme heat or cold, drought or flooding, nutrient deficiencies, wounding, and intense light, as well as biotic stresses, such as pathogen attack, also affect the senescence time. Even though extensive studies have discussed the complex regulatory mechanisms underlying leaf senescence, whole-plant senescence, which is the definite last stage in a plant’s life, has not been well investigated. Future studies are needed to deepen our understanding of the epigenetic mechanisms behind regulating stress-triggered and developmental senescence. In addition, focusing on understanding the molecular mechanisms underlying the epigenetic regulation of whole-plant senescence could reveal senescence-specific epigenetic mechanisms for plants, and could hopefully distinguish between leaf senescence and whole-plant senescence regulation. Future studies seeking answers to the questions, such as the following, are encouraged: (1) Is there a difference between senescence upregulated genes and senescence downregulated genes in terms of epigenetic regulation? (2) Which epigenetic mechanism is more involved in senescence? (3) Is there a difference between the epigenetic regulation of stress-induced vs. age-dependent senescence? In addition, as most senescence studies have focused on *Arabidopsis*, by studying other plant species, one could investigate (4) if epigenetic mechanisms of senescence may be conserved among plant species.

## References

[1] Guo Y., Gan S. (2005). Leaf senescence: Signals, execution, and regulation. Curr. Top. Dev. Biol..

[2] Yolcu S., Li X., Li S., Kim Y.J. (2018). Beyond the genetic code in leaf senescence. J. Exp. Bot..

[3] Woo H.R., Kim H.J., Nam H.G., Lim P.O. (2013). Plant leaf senescence and death—Regulation by multiple layers of control and implications for aging in general. J. Cell Sci..

[4] Breeze E., Harrison E., McHattie S., Hughes L., Hickman R., Hill C., Kiddle S., Kim Y.S., Penfold C.A., Jenkins D. (2011). High-resolution temporal profiling of transcripts during Arabidopsis leaf senescence reveals a distinct chronology of processes and regulation. Plant Cell.

[5] Woo H.R., Koo H.J., Kim J., Jeong H., Yang J.O., Lee I.H., Jun J.H., Choi S.H., Park S.J., Kang B. (2016). Programming of Plant Leaf Senescence with Temporal and Inter-Organellar Coordination of Transcriptome in Arabidopsis. Plant Physiol..

[6] Hinderhofer K., Zentgraf U. (2001). Identification of a transcription factor specifically expressed at the onset of leaf senescence. Planta.

[7] Humbeck K. (2013). Epigenetic and small RNA regulation of senescence. Plant Mol. Biol..

[8] Stroud H., Greenberg M.V., Feng S., Bernatavichute Y.V., Jacobsen S.E. (2013). Comprehensive analysis of silencing mutants reveals complex regulation of the Arabidopsis methylome. Cell.

[9] Ay N., Janack B., Humbeck K. (2014). Epigenetic control of plant senescence and linked processes. J. Exp. Bot..

[10] Miryeganeh M. (2021). Senescence: The Compromised Time of Death That Plants May Call on Themselves. Genes.

[11] Vanyushin B.F., Ashapkin V.V. (2011). DNA methylation in higher plants: Past, present and future. Biochim. Biophys. Acta.

[12] Choi Y., Gehring M., Johnson L., Hannon M., Harada J.J., Goldberg R.B., Jacobsen S.E., Fischer R.L. (2002). DEMETER, a DNA glycosylase domain protein, is required for endosperm gene imprinting and seed viability in arabidopsis. Cell.

[13] Gehring M., Reik W., Henikoff S. (2009). DNA demethylation by DNA repair. Trends Genet..

[14] Inamdar N.M., Ehrlich K.C., Ehrlich M. (1991). CpG methylation inhibits binding of several sequence-specific DNA-binding proteins from pea, wheat, soybean and cauliflower. Plant Mol. Biol..

[15] Chan S.W., Henderson I.R., Jacobsen S.E. (2005). Gardening the genome: DNA methylation in Arabidopsis thaliana. Nat. Rev. Genet..

[16] Monteuuis O., Doulbeau S., Verdeil J.-L. (2008). DNA methylation in different origin clonal offspring from a mature Sequoiadendron giganteum genotype. Trees.

[17] Ostrowska-Mazurek A., Kasprzak P., Kubala S., Zaborowska M., Sobieszczuk-Nowicka E. (2020). Epigenetic Landmarks of Leaf Senescence and Crop Improvement. Int. J. Mol. Sci..

[18] Li H., Freeling M., Lisch D. (2010). Epigenetic reprogramming during vegetative phase change in maize. Proc. Natl. Acad. Sci. USA.

[19] Kim M., Ohr H., Lee J.W., Hyun Y., Fischer R.L., Choi Y. (2008). Temporal and spatial downregulation of Arabidopsis MET1 activity results in global DNA hypomethylation and developmental defects. Mol. Cells.

[20] Horváth V., Merenciano M., González J. (2017). Revisiting the Relationship between Transposable Elements and the Eukaryotic Stress Response. Trends Genet..

[21] Groth M., Moissiard G., Wirtz M., Wang H., Garcia-Salinas C., Ramos-Parra P.A., Bischof S., Feng S., Cokus S.J., John A. (2016). MTHFD1 controls DNA methylation in Arabidopsis. Nat. Commun..

[22] Guo Y., Gan S.-S. (2012). Convergence and divergence in gene expression profiles induced by leaf senescence and 27 senescence-promoting hormonal, pathological and environmental stress treatments. Plant Cell Environ..

[23] Ay N., Clauß K., Barth O., Humbeck K. (2008). Identification and characterization of novel senescence-associated genes from barley (Hordeum vulgare) primary leaves. Plant Biol..

[24] He L., Wu W., Zinta G., Yang L., Wang D., Liu R., Zhang H., Zheng Z., Huang H., Zhang Q. (2018). A naturally occurring epiallele associates with leaf senescence and local climate adaptation in Arabidopsis accessions. Nat. Commun..

[25] Lambé P., Mutambel H.S.N., Fouché J.-G., Deltour R., Foidart J.-M., Gaspar T. (1997). DNA methylation as a key process in regulation of organogenic totipotency and plant neoplastic progression?. In Vitro Cell. Dev. Biol.—Plant.

[26] Trejo-Arellano M.S., Mehdi S., de Jonge J., Dvorák Tomastíková E., Köhler C., Hennig L. (2020). Dark-Induced Senescence Causes Localized Changes in DNA Methylation1. Plant Physiol..

[27] Vatov E., Zentgraf U., Ludewig U. (2021). Moderate DNA methylation changes associated with nitrogen remobilization and leaf senescence in Arabidopsis. bioRxiv.

[28] Greenwood M.S. (1995). Juvenility and maturation in conifers: Current concepts. Tree Physiol..

[29] Galaud J.-P., Gaspar T., Boyer N. (1993). Effect of anti-DNA methylation drugs on growth, level of methylated DNA, peroxidase activity and ethylene production of Bryonia dioica internodes. Physiol. Plant..

[30] Fraga M.F., Rodríguez R., Cañal M.J. (2002). Genomic DNA methylation-demethylation during aging and reinvigoration of Pinus radiata. Tree Physiol..

[31] Ogneva Z.V., Dubrovina A.S., Kiselev K.V. (2016). Age-associated alterations in DNA methylation and expression of methyltransferase and demethylase genes in Arabidopsis thaliana. Biol. Plant..

[32] Yuan L., Wang D., Cao L., Yu N., Liu K., Guo Y., Gan S., Chen L. (2020). Regulation of Leaf Longevity by DML3-Mediated DNA Demethylation. Mol. Plant.

[33] Luger K., Mäder A.W., Richmond R.K., Sargent D.F., Richmond T.J. (1997). Crystal structure of the nucleosome core particle at 2.8 A resolution. Nature.

[34] Strahl B.D., Allis C.D. (2000). The language of covalent histone modifications. Nature.

[35] Jenuwein T., Allis C.D. (2001). Translating the histone code. Science.

[36] Kouzarides T. (2007). Chromatin modifications and their function. Cell.

[37] Pandey R., Müller A., Napoli C.A., Selinger D.A., Pikaard C.S., Richards E.J., Bender J., Mount D.W., Jorgensen R.A. (2002). Analysis of histone acetyltransferase and histone deacetylase families of Arabidopsis thaliana suggests functional diversification of chromatin modification among multicellular eukaryotes. Nucleic Acids Res..

[38] Guo Y., Ren G., Zhang K., Li Z., Miao Y., Guo H. (2021). Leaf senescence: Progression, regulation, and application. Mol. Hortic..

[39] Ay N., Irmler K., Fischer A., Uhlemann R., Reuter G., Humbeck K. (2009). Epigenetic programming via histone methylation at WRKY53 controls leaf senescence in Arabidopsis thaliana. Plant J..

[40] Brusslan J.A., Bonora G., Rus-Canterbury A.M., Tariq F., Jaroszewicz A., Pellegrini M. (2015). A Genome-Wide Chronological Study of Gene Expression and Two Histone Modifications, H3K4me3 and H3K9ac, during Developmental Leaf Senescence. Plant Physiol..

[41] Naumann K., Fischer A., Hofmann I., Krauss V., Phalke S., Irmler K., Hause G., Aurich A.C., Dorn R., Jenuwein T. (2005). Pivotal role of AtSUVH2 in heterochromatic histone methylation and gene silencing in Arabidopsis. EMBO J..

[42] Jing Y., Sun H., Yuan W., Wang Y., Li Q., Liu Y., Li Y., Qian W. (2016). SUVH2 and SUVH9 Couple Two Essential Steps for Transcriptional Gene Silencing in Arabidopsis. Mol. Plant.

[43] Brusslan J.A., Rus Alvarez-Canterbury A.M., Nair N.U., Rice J.C., Hitchler M.J., Pellegrini M. (2012). Genome-wide evaluation of histone methylation changes associated with leaf senescence in Arabidopsis. PLoS ONE.

[44] Weaver L.M., Gan S., Quirino B., Amasino R.M. (1998). A comparison of the expression patterns of several senescence-associated genes in response to stress and hormone treatment. Plant Mol. Biol..

[45] Yan H., Liu Y., Zhang K., Song J., Xu W., Su Z. (2019). Chromatin State-Based Analysis of Epigenetic H3K4me3 Marks of Arabidopsis in Response to Dark Stress. Front. Genet..

[46] Liebsch D., Keech O. (2016). Dark-induced leaf senescence: New insights into a complex light-dependent regulatory pathway. New Phytol..

[47] Liu P., Zhang S., Zhou B., Luo X., Zhou X.F., Cai B., Jin Y.H., Niu D., Lin J., Cao X. (2019). The Histone H3K4 Demethylase JMJ16 Represses Leaf Senescence in Arabidopsis. Plant Cell.

[48] Wang X., Gao J., Gao S., Song Y., Yang Z., Kuai B. (2019). The H3K27me3 demethylase REF6 promotes leaf senescence through directly activating major senescence regulatory and functional genes in Arabidopsis. PLoS Genet..

[49] Lu F., Cui X., Zhang S., Jenuwein T., Cao X. (2011). Arabidopsis REF6 is a histone H3 lysine 27 demethylase. Nat. Genet..

[50] Verdin E., Ott M. (2015). 50 years of protein acetylation: From gene regulation to epigenetics, metabolism and beyond. Nat. Rev. Mol. Cell Biol..

[51] Chen X., Lu L., Mayer K.S., Scalf M., Qian S., Lomax A., Smith L.M., Zhong X. (2016). POWERDRESS interacts with HISTONE DEACETYLASE 9 to promote aging in Arabidopsis. eLife.

[52] Zhu M., Li Y., Chen G., Ren L., Xie Q., Zhao Z., Hu Z. (2015). Silencing SlELP2L, a tomato Elongator complex protein 2-like gene, inhibits leaf growth, accelerates leaf, sepal senescence, and produces dark-green fruit. Sci. Rep..

[53] Huang D., Lan W., Li D., Deng B., Lin W., Ren Y., Miao Y. (2018). WHIRLY1 Occupancy Affects Histone Lysine Modification and WRKY53 Transcription in Arabidopsis Developmental Manner. Front. Plant Sci..

[54] Hinckley W.E., Keymanesh K., Cordova J.A., Brusslan J.A. (2019). The HAC1 histone acetyltransferase promotes leaf senescence and regulates the expression of ERF022. Plant Direct..

[55] Hu Y., Lu Y., Zhao Y., Zhou D.X. (2019). Histone Acetylation Dynamics Integrates Metabolic Activity to Regulate Plant Response to Stress. Front. Plant Sci..

[56] Ueda M., Seki M. (2020). Histone Modifications Form Epigenetic Regulatory Networks to Regulate Abiotic Stress Response. Plant Physiol..

[57] Tian L., Chen Z.J. (2001). Blocking histone deacetylation in Arabidopsis induces pleiotropic effects on plant gene regulation and development. Proc. Natl. Acad. Sci. USA.

[58] Wu K., Zhang L., Zhou C., Yu C.W., Chaikam V. (2008). HDA6 is required for jasmonate response, senescence and flowering in Arabidopsis. J. Exp. Bot..

[59] Kim J., Kim J.H., Lyu J.I., Woo H.R., Lim P.O. (2018). New insights into the regulation of leaf senescence in Arabidopsis. J. Exp. Bot..

[60] Kuang J.F., Chen J.Y., Luo M., Wu K.Q., Sun W., Jiang Y.M., Lu W.J. (2012). Histone deacetylase HD2 interacts with ERF1 and is involved in longan fruit senescence. J. Exp. Bot..

[61] Han S.K., Wu M.F., Cui S., Wagner D. (2015). Roles and activities of chromatin remodeling ATPases in plants. Plant J..

[62] Kusch T., Workman J.L. (2007). Histone variants and complexes involved in their exchange. Subcell Biochem..

[63] Chodavarapu R.K., Feng S., Bernatavichute Y.V., Chen P.Y., Stroud H., Yu Y., Hetzel J.A., Kuo F., Kim J., Cokus S.J. (2010). Relationship between nucleosome positioning and DNA methylation. Nature.

[64] Berr A., Shafiq S., Shen W.-H. (2011). Histone modifications in transcriptional activation during plant development. Biochim. Biophys. Acta (BBA)—Gene Regul. Mech..

[65] Miryeganeh M. (2021). Plants’ Epigenetic Mechanisms and Abiotic Stress. Genes.

[66] Pradhan B., Jangid K.K., Sarwat M., Bishi S.K., Sarwat M., Tuteja N. (2019). Chapter 11—Role of Histones During Leaf Senescence. Senescence Signalling and Control in Plants.

[67] Lim P.O., Kim Y., Breeze E., Koo J.C., Woo H.R., Ryu J.S., Park D.H., Beynon J., Tabrett A., Buchanan-Wollaston V. (2007). Overexpression of a chromatin architecture-controlling AT-hook protein extends leaf longevity and increases the post-harvest storage life of plants. Plant J..

[68] Li C., Gu L., Gao L., Chen C., Wei C.-Q., Qiu Q., Chien C.-W., Wang S., Jiang L., Ai L.-F. (2016). Concerted genomic targeting of H3K27 demethylase REF6 and chromatin-remodeling ATPase BRM in Arabidopsis. Nat. Genet..

[69] Efroni I., Han S.K., Kim H.J., Wu M.F., Steiner E., Birnbaum K.D., Hong J.C., Eshed Y., Wagner D. (2013). Regulation of leaf maturation by chromatin-mediated modulation of cytokinin responses. Dev. Cell.

[70] Archacki R., Yatusevich R., Buszewicz D., Krzyczmonik K., Patryn J., Iwanicka-Nowicka R., Biecek P., Wilczynski B., Koblowska M., Jerzmanowski A. (2017). Arabidopsis SWI/SNF chromatin remodeling complex binds both promoters and terminators to regulate gene expression. Nucleic Acids Res..

[71] Cho E.J., Choi S.H., Kim J.H., Kim J.E., Lee M.H., Chung B.Y., Woo H.R., Kim J.H. (2016). A Mutation in Plant-Specific SWI2/SNF2-Like Chromatin-Remodeling Proteins, DRD1 and DDM1, Delays Leaf Senescence in Arabidopsis thaliana. PLoS ONE.

[72] van der Graaff E., Schwacke R., Schneider A., Desimone M., Flügge U.-I., Kunze R. (2006). Transcription Analysis of Arabidopsis Membrane Transporters and Hormone Pathways during Developmental and Induced Leaf Senescence. Plant Physiol..

[73] Schippers J.H.M., Nunes-Nesi A., Apetrei R., Hille J., Fernie A.R., Dijkwel P.P. (2008). The Arabidopsis onset of leaf death5 Mutation of Quinolinate Synthase Affects Nicotinamide Adenine Dinucleotide Biosynthesis and Causes Early Ageing. Plant Cell.

[74] Balazadeh S., Siddiqui H., Allu A.D., Matallana-Ramirez L.P., Caldana C., Mehrnia M., Zanor M.I., Köhler B., Mueller-Roeber B. (2010). A gene regulatory network controlled by the NAC transcription factor ANAC092/AtNAC2/ORE1 during salt-promoted senescence. Plant J..

[75] Fernández-Calvino L., Guzmán-Benito I., Del Toro F.J., Donaire L., Castro-Sanz A.B., Ruíz-Ferrer V., Llave C. (2016). Activation of senescence-associated Dark-inducible (DIN) genes during infection contributes to enhanced susceptibility to plant viruses. Mol. Plant Pathol..

[76] Sakuraba Y., Jeong J., Kang M.Y., Kim J., Paek N.C., Choi G. (2014). Phytochrome-interacting transcription factors PIF4 and PIF5 induce leaf senescence in Arabidopsis. Nat. Commun..

[77] Pecinka A., Dinh H.Q., Baubec T., Rosa M., Lettner N., Mittelsten Scheid O. (2010). Epigenetic regulation of repetitive elements is attenuated by prolonged heat stress in Arabidopsis. Plant Cell.

[78] Knoth C., Ringler J., Dangl J.L., Eulgem T. (2007). Arabidopsis WRKY70 is required for full RPP4-mediated disease resistance and basal defense against Hyaloperonospora parasitica. Mol. Plant Microbe Interact..

[79] Alvarez-Venegas R., Abdallat A.A., Guo M., Alfano J.R., Avramova Z. (2007). Epigenetic control of a transcription factor at the cross section of two antagonistic pathways. Epigenetics.

[80] Besseau S., Li J., Palva E.T. (2012). WRKY54 and WRKY70 co-operate as negative regulators of leaf senescence in Arabidopsis thaliana. J. Exp. Bot..

[81] Hu Y., Dong Q., Yu D. (2012). Arabidopsis WRKY46 coordinates with WRKY70 and WRKY53 in basal resistance against pathogen Pseudomonas syringae. Plant Sci..

[82] Alvarez-Venegas R., Avramova Z. (2005). Methylation patterns of histone H3 Lys 4, Lys 9 and Lys 27 in transcriptionally active and inactive Arabidopsis genes and in atx1 mutants. Nucleic Acids Res..

[83] Kim J.M., To T.K., Ishida J., Matsui A., Kimura H., Seki M. (2012). Transition of chromatin status during the process of recovery from drought stress in Arabidopsis thaliana. Plant Cell Physiol..

[84] Janack B., Sosoi P., Krupinska K., Humbeck K. (2016). Knockdown of WHIRLY1 Affects Drought Stress-Induced Leaf Senescence and Histone Modifications of the Senescence-Associated Gene HvS40. Plants.

[85] Mengel A., Ageeva A., Georgii E., Bernhardt J., Wu K., Durner J., Lindermayr C. (2017). Nitric Oxide Modulates Histone Acetylation at Stress Genes by Inhibition of Histone Deacetylases. Plant Physiol..

[86] Chen L.T., Luo M., Wang Y.Y., Wu K. (2010). Involvement of Arabidopsis histone deacetylase HDA6 in ABA and salt stress response. J. Exp. Bot..

[87] Chen L.T., Wu K. (2010). Role of histone deacetylases HDA6 and HDA19 in ABA and abiotic stress response. Plant Signal. Behav..

[88] Becker W., Apel K. (1993). Differences in gene expression between natural and artificially induced leaf senescence. Planta.

[89] Mehta A., Haber J.E. (2014). Sources of DNA double-strand breaks and models of recombinational DNA repair. Cold Spring Harb. Perspect. Biol..

[90] Tuteja N., Singh M.B., Misra M.K., Bhalla P.L., Tuteja R. (2001). Molecular mechanisms of DNA damage and repair: Progress in plants. Crit. Rev. Biochem. Mol. Biol..

[91] White R.R., Vijg J. (2016). Do DNA Double-Strand Breaks Drive Aging?. Mol. Cell.

[92] Waterworth W.M., Footitt S., Bray C.M., Finch-Savage W.E., West C.E. (2016). DNA damage checkpoint kinase ATM regulates germination and maintains genome stability in seeds. Proc. Natl. Acad. Sci. USA.

[93] Britt A.B. (1996). DNA damage and repair in plants. Annu. Rev. Plant Physiol. Plant Mol. Biol..

[94] Culligan K.M., Robertson C.E., Foreman J., Doerner P., Britt A.B. (2006). ATR and ATM play both distinct and additive roles in response to ionizing radiation. Plant J..

[95] Yoshiyama K., Conklin P.A., Huefner N.D., Britt A.B. (2009). Suppressor of gamma response 1 (SOG1) encodes a putative transcription factor governing multiple responses to DNA damage. Proc. Natl. Acad. Sci. USA.

[96] Du L.L., Nakamura T.M., Russell P. (2006). Histone modification-dependent and -independent pathways for recruitment of checkpoint protein Crb2 to double-strand breaks. Genes Dev..

[97] Drury G.E., Dowle A.A., Ashford D.A., Waterworth W.M., Thomas J., West C.E. (2012). Dynamics of plant histone modifications in response to DNA damage. Biochem. J..

[98] Li Z., Kim J.H., Kim J., Lyu J.I., Zhang Y., Guo H., Nam H.G., Woo H.R. (2020). ATM suppresses leaf senescence triggered by DNA double-strand break through epigenetic control of senescence-associated genes in Arabidopsis. New Phytol..

[99] Freitas A.A., de Magalhães J.P. (2011). A review and appraisal of the DNA damage theory of ageing. Mutat. Res..

[100] Pulido A., Laufs P. (2010). Co-ordination of developmental processes by small RNAs during leaf development. J. Exp. Bot..

[101] Rubio-Somoza I., Weigel D. (2011). MicroRNA networks and developmental plasticity in plants. Trends Plant Sci..

[102] Wu L., Zhou H., Zhang Q., Zhang J., Ni F., Liu C., Qi Y. (2010). DNA methylation mediated by a microRNA pathway. Mol. Cell.

[103] Ito H. (2012). Small RNAs and transposon silencing in plants. Dev. Growth Differ..

[104] Kim J.H., Woo H.R., Kim J., Lim P.O., Lee I.C., Choi S.H., Hwang D., Nam H.G. (2009). Trifurcate feed-forward regulation of age-dependent cell death involving miR164 in Arabidopsis. Science.

[105] Schommer C., Palatnik J.F., Aggarwal P., Chételat A., Cubas P., Farmer E.E., Nath U., Weigel D. (2008). Control of Jasmonate Biosynthesis and Senescence by miR319 Targets. PLoS Biol..

[106] Debernardi J.M., Mecchia M.A., Vercruyssen L., Smaczniak C., Kaufmann K., Inze D., Rodriguez R.E., Palatnik J.F. (2014). Post-transcriptional control of GRF transcription factors by microRNA miR396 and GIF co-activator affects leaf size and longevity. Plant J..

[107] Lim P.O., Lee I.C., Kim J., Kim H.J., Ryu J.S., Woo H.R., Nam H.G. (2010). Auxin response factor 2 (ARF2) plays a major role in regulating auxin-mediated leaf longevity. J. Exp. Bot..

[108] Ellis C.M., Nagpal P., Young J.C., Hagen G., Guilfoyle T.J., Reed J.W. (2005). AUXIN RESPONSE FACTOR1 and AUXIN RESPONSE FACTOR2regulate senescence and floral organ abscission in Arabidopsis thaliana. Development.

[109] Arikit S., Xia R., Kakrana A., Huang K., Zhai J., Yan Z., Valdés-López O., Prince S., Musket T.A., Nguyen H.T. (2014). An atlas of soybean small RNAs identifies phased siRNAs from hundreds of coding genes. Plant Cell.

[110] Marin E., Jouannet V., Herz A., Lokerse A.S., Weijers D., Vaucheret H., Nussaume L., Crespi M.D., Maizel A. (2010). miR390, Arabidopsis TAS3 tasiRNAs, and their AUXIN RESPONSE FACTOR targets define an autoregulatory network quantitatively regulating lateral root growth. Plant Cell.

[111] Luan W., Dai Y., Li X.-Y., Wang Y., Tao X., Li C.-X., Mao P., Ma X.-R. (2020). Identification of tRFs and phasiRNAs in tomato (Solanum lycopersicum) and their responses to exogenous abscisic acid. BMC Plant Biol..

[112] Miryeganeh M. (2020). Synchronization of senescence and desynchronization of flowering in *Arabidopsis thaliana*. AoB Plants.

[113] Tariq M., Paszkowski J. (2004). DNA and histone methylation in plants. Trends Genet..

[114] Saze H., Tsugane K., Kanno T., Nishimura T. (2012). DNA methylation in plants: Relationship to small RNAs and histone modifications, and functions in transposon inactivation. Plant Cell Physiol..

[115] Miryeganeh M., Saze H. (2020). Epigenetic inheritance and plant evolution. Popul. Ecol..

